# From Channels to Canonical Wnt Signaling: A Pathological Perspective

**DOI:** 10.3390/ijms22094613

**Published:** 2021-04-28

**Authors:** Silvia Muccioli, Valentina Brillo, Leonardo Chieregato, Luigi Leanza, Vanessa Checchetto, Roberto Costa

**Affiliations:** Department of Biology, University of Padova, 35122 Padova, Italy; silvia.muccioli@studenti.unipd.it (S.M.); valentina.brillo@studenti.unipd.it (V.B.); leonardo.chieregato@studenti.unipd.it (L.C.); vanessa.checchetto@unipd.it (V.C.); roberto.costa@unipd.it (R.C.)

**Keywords:** Wnt signaling, ion channels, cancer, metabolic diseases, neurological disorders

## Abstract

Wnt signaling is an important pathway mainly active during embryonic development and controlling cell proliferation. This regulatory pathway is aberrantly activated in several human diseases. Ion channels are known modulators of several important cellular functions ranging from the tuning of the membrane potential to modulation of intracellular pathways, in particular the influence of ion channels in Wnt signaling regulation has been widely investigated. This review will discuss the known links between ion channels and canonical Wnt signaling, focusing on their possible roles in human metabolic diseases, neurological disorders, and cancer.

## 1. Introduction

The Wnt signaling pathway is a group of signaling transduction cascades involved in various physiological processes, including embryonic development, cell stemness maintenance, and tissue regeneration [1]. The term “Wnt” is the combination of two gene annotations, coding homologous proteins in *D. melanogaster* and *M. musculus*, respectively *wingless* (*wg*) and *Mammary tumor virus integration 1(Int1*) [2].

Currently, three classes of Wnt signaling pathways have been reported: (i) the canonical Wnt pathway, (ii) the non-canonical planar cell polarity pathway, and (iii) the non-canonical Wnt/calcium pathway [1]. All pathways share a similar initiation of the cascade due to the binding of a Wnt ligand to a Frizzled family receptor. Wnt ligands are small secreted proteins able to diffuse in the extracellular matrix, generating a response paracrine gradient.

**Figure 1:**
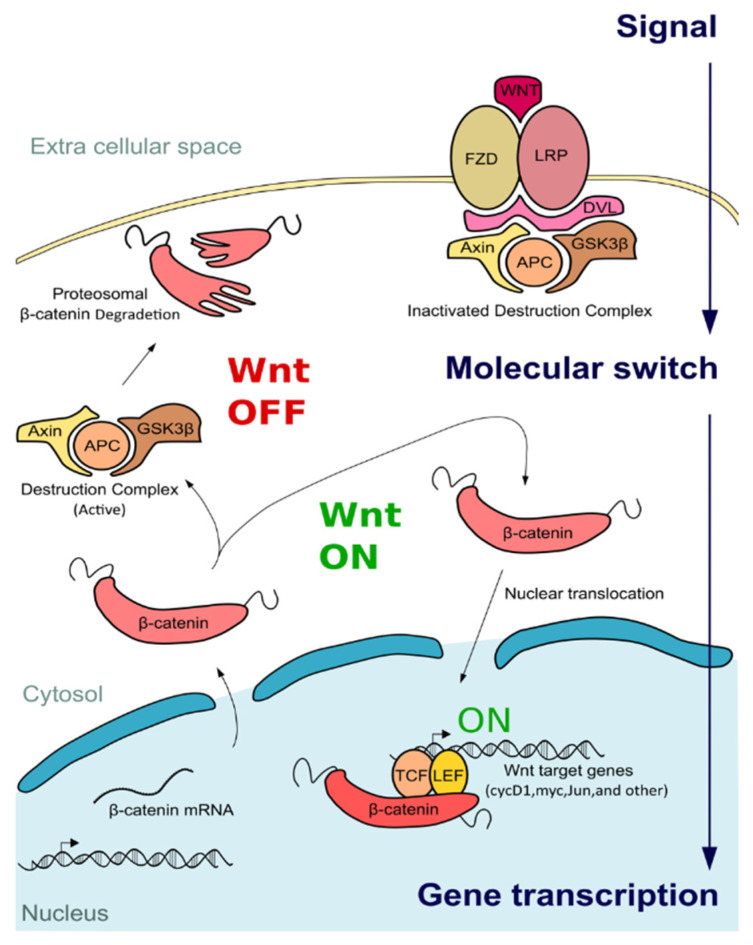
Canonical Wnt signaling cascade. Generally, Wnt signaling is “OFF” since without any stimulus, β-catenin is degraded by the proteasome after phosphorylation mediated by the destruction complex (including Axin1 (Axin), Adenomatous polyposis coli (APC), and the glycogen synthase kinase 3β (GSK3β)). Conversely, when Wnt ligands bind to the receptors Frizzled (FZD) and Lipoprotein receptor-related protein 5/6 (LRP), Dishevelled (DvI) is recruited and contributes to the destruction complex inactivation. Consequently, β-catenin is not restricted and can translocate into the nucleus contributing to the activation of the transcription of Wnt-related genes, directly binding the T cell lymphocyte differentiation factors (TCF) and lymphoid enhancer-binding factor 1 (LEF).

The binding of a Wnt ligand triggers canonical Wnt signaling (Figure 1) (e.g., WNT3A) with Frizzled (FZD) and Lipoprotein receptor-related protein 5/6 (LRP5/6), forming a complex [3]. The intracellular domain of this active complex transduces the stimulus inside the cell, inducing the crucial interactor Dishevelled (DVL) recruitment. DVL creates an extensive scaffold for other proteins, including Axin1, Adenomatous polyposis coli (APC), and the glycogen synthase kinase 3β (GSK3β), three negative regulators of the β-catenin, also known as the β-catenin destruction complex [4,5]. Indeed, since β-catenin is constitutively transcribed, it must be constantly controlled at the post-translational level by this destruction machinery, involving β-Transducin Repeat Containing E3 Ubiquitin Protein Ligase and the proteasome [6].

On the other end, the inhibition of GSK3β along with the sequestering of Axin1 and APC results in the impairment of the β-catenin destruction complex and the activation of Wnt pathway [7]. Once activated, β-catenin is stabilized, and its concentration increases over the threshold level so that it translocates across the nuclear envelope and acts as a co-transcriptional regulator.

In general, β-catenin indirectly enables the interaction between lymphoid enhancer-binding factor 1 (LEF1), the T cell lymphocyte differentiation factors (TCFs) and the Wnt responsive element, short DNA sequences located in the promoters of Wnt target genes [1]; however, the transcriptional activation involves the release of Groucho, other repressors, and chromatin remodeling proteins from TCFs [8,9,10]. This simple event switches the function of TCFs [8], which stops the recruitment of histone deacetylases [8], [11] and primes the association between β-catenin, RNA polymerase II, and a plethora of transcriptional factors, including CREB binding protein (CBP) and mediator (MED) [12,13]. The intra-nuclear canonical Wnt signaling phases are a very complex network of interactions (further details about this topic can be found in Anthony et al., 2020 [14]), and may involve unusual partners, which may even revert β-catenin activity, blocking Wnt mediated transcription. For instance, Hypoxia Inducible Factor 1α, and several Sry-related HMG box proteins can bind β-catenin, hijacking its activity [14,15,16,17].

In parallel with the role of the transcriptional activator, the Wnt/ β-catenin cascade can induce huge changes in cytoskeleton remodeling and endosome dynamics [18,19,20], altering cell polarity, cell adhesion, proliferation, and migration. Indeed, catenins (α-catenin and β-catenin) were initially identified as structural proteins involved in cytoskeleton formation and cell/cell adhesion due to their engagement in cadherin/F-Actin complexes [21,22]. Altogether, β-catenin mediated transcriptional activity and release by the cytoskeleton contribute to the whole response of the canonical cascade, affecting the cell physiology and inducing epidermal to mesenchymal transition (EMT) [23].

Among the three different Wnt signaling pathways, the canonical (β-catenin-dependent) one is better described in human diseases. Aberrant regulation of this molecular cascade triggers catastrophic consequences and is related to the insurgence of many human pathologies, including cancer and human birth disorders [24,25,26,27]. Canonical Wnt signaling is directly and indirectly modulated by a multitude of different stimuli and events. This form of integration of the signal generates a hub in cellular signaling that finely coordinates cell proliferation, differentiation, and gene expression [1]. In addition, calcium (Ca^2+^) signaling, endoplasmic reticulum (ER) stress, and mitochondrial cation homeostasis have been recently related to Wnt regulation, revealing a new connection between ion fluxes, channels, and Wnt-dependent cellular signaling [28].

Even more, the non-canonical Wnt Ca^2+^ pathway further complicates this network by its ability in cytoplasmic Ca^2+^ handling. The ligand WNT5 induces the formation of the ROR2-FZD complex, which in turn activates phospholipase C. This event causes the release of the ER-stored Ca^2+^ by inositol-1,4,5-triphosphate (IP_3_) dependent channel activity and the activation of NFAT signaling cascade by the kinase PKC [29]. In addition, non-canonical Wnt Ca^2+^ pathway can also tune canonical Wnt signaling [30]. The detrimental action of canonical Wnt signaling in the pathologic context can be related to the role of β-catenin in the regulation of gene transcription and its role in EMT. Indeed, more than three hundred genes seem to be positively regulated by this transcription factor [31]. For example, the cyclin D1 (*CCND1*) and the protoncogene myc (*MYC*) are up-regulated by β-catenin, thus inducing G1 to the S phase cell cycle transition. Since this last step is characterized by DNA replication, it ultimately leads to mitosis and cell proliferation [32,33].

## 2. Molecular Interplay between Ions, Channels and Wnt Signaling

The crucial node of cellular signaling is the coordination and the unambiguous transmission of the signal. Cells usually succeed in this task by integrating several stimuli, ensuring the precise signal in the proper cell type, according to the right spatio-temporal frame. Channel proteins, ions, and secondary messengers are crucial mediators in this field, influencing both the cytosolic and the nuclear stages of Wnt signaling. Concerning molecular mechanisms in which ions and channels tune the canonical Wnt, we recapitulate four synergic mechanisms proposed by different research groups (Ca^2+^ mediated β-catenin nuclear translocation; Ca^2+^ storage in the ER modulates ER stress mediated Wnt repression; Channels modulate PI3K/AKT cascade and indirectly the canonical Wnt Axis; Aquaporin1 (AQP1) physically participates at the formation of the β-catenin destruction complex). These events control distinct steps of the canonical cascade, and such stimuli contribute to the whole transduction process in a coordinated fashion, supporting each other in regulating the cellular response.

**Figure 2:**
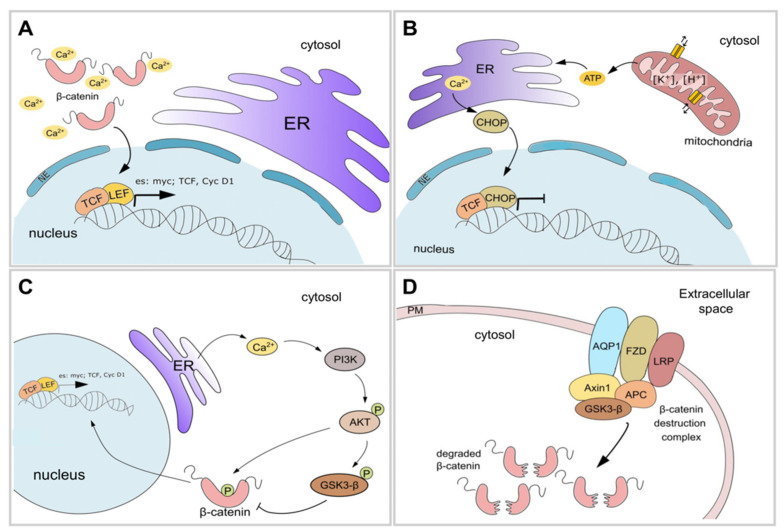
Ca^2+^ dynamics finely tune canonical Wnt signaling. (**A**) β-catenin is a negatively charged protein, which cannot passively overcome nuclear membranes. Ca^2+^ release from the ER contributes to the neutralization of the negatively charged residues of β-catenin, enabling its translocation into the nucleus. (**B**) Mitochondrial homeostasis are requirements for all the cell, including glycolytic ones. Indeed, the mitochondrial ATP synthesis enables Ca^2+^ storage in the ER. When the ion balance (e.g., [H^+^], [K^+^] and/or [Ca^2+^]) is improper, the ER-stress arise. The ER-stress cascade activates CHOP, which represses Wnt signaling by competing with TCF, ultimately resulting in negative regulation of the Wnt cascade. (**C**) Wnt signaling is closely interconnected with other pathways, such as the PI3K/AKT pathway, which plays a crucial role in Wnt signaling regulation by modulating GSK3β activity. In this context, Ca^2+^ concentration plays a critical role in the Wnt/AKT crosstalk. (**D**) Channel proteins can also contribute to Wnt regulation in a pore-independent manner, such as *Aquaporin1* (AQP1). Recent evidence demonstrated that AQP1 physically interacts with GSK3β, Axin1, and LRP in a multi-complex platform that eventually promotes complex activity destruction.

Ca^2+^ dynamics finely modulate the β-catenin nuclear translocation. Ca^2+^ is a secondary cellular messenger, regulating many proteins and biological events, including Wnt mediators. The activation of ER Ca^2+^ channels (e.g., IP3 receptor Ryanodine receptors, and Orai calcium channels) allows local and transient fluxes of Ca^2+^, which can perturbate the basal Ca^2+^ level in the cytosol (~100 nM), generating a sharp and transient increase in Ca^2+^ concentration (>2 µM) [34]. Moreover, Ca^2+^ is a highly reactive chemical species inside the cell, as it binds acidic residues located on the protein surface, tuning their activities and influencing their interaction with other molecules. On the other hand, β-catenin is a negatively charged nuclear transcription factor abundant in the cytosol, which cannot passively overcome nuclear membranes, thus ensuring the unambiguous activation of the Wnt pathway. Ca^2+^ release from the ER contributes to the neutralization of the negatively charged residues of β-catenin (Figure 2A), enabling its translocation into the nucleus [35], where it can bind its responsive elements located in the Wnt-dependent promoters.

A synergic mechanism able to modulate canonical Wnt signaling (independently to the previously described) involves the Ca^2+^ concentration in the ER, maintained by sarco-endoplasmic reticulum calcium ATPase (SERCA) activity, and the ATP/ADP fluxes between mitochondria and ER are critical determinants in the Wnt response through ER stress level [17,36]. Thus, the Krebs cycle modulation or the perturbation of potassium (K^+^) and proton gradient can induce intracellular ER stress development, resulting in increased CHOP (also known as GADD153 or DDIT3) protein levels. CHOP represses Wnt signaling by competing with TCF, the final effector of canonical Wnt signaling cascade, ultimately resulting in negative regulation of the proliferation rate (Figure 2B) [37,38]. Ion balance also contributes to the whole signaling, coordinating different transduction cascades involved in cell proliferation. Several independent authors demonstrated that K^+^ homeostasis can also impact the canonical Wnt signaling. Concerning this point, (i) Koval and colleagues showed that clofazimine, an inhibitor of the K^+^ channel Kv1.3 [39], affects the Wnt signaling and cancer proliferation in triple-negative breast cancer (TNBC) [40]; (ii) Breuer et al. demonstrate that NS1643, a compound able to activate the Kv11.1 channel, attenuates metastasis spread and Wnt signaling in TNBC [41]; (iii) salinomycin, a K^+^ ionophore [42], downregulates the canonical Wnt signaling in chronic lymphocytic leukemia cells and colon cancer cells, similarly to nigericin, ionomycin, and thapsigargin, suggesting that the whole ions balance is involved in this control [36,43].

Wnt signaling is also closely interconnected with other pathways, such as the PI3K/AKT pathway, which plays a crucial role in Wnt signaling regulation by modulating GSK3β activity. Moreover, AKT is a master regulator of a plethora of cellular physiological events, such as apoptosis, proliferation, metabolic reprogramming, and cell signaling tuning, which, if dysregulated, results in the emergence of several disorders [44]. In this context, Ca^2+^ concentration plays a critical role in the Wnt/AKT crosstalk [45]. Thus, the activation of the IP3 receptor induces cytosolic Ca^2+^ release, which activates PI3K and AKT. Subsequently, AKT both inactivates GSK3β by phosphorylation and promotes β-catenin activity through direct phosphorylation of Ser-552 in the activation domain. These reactions eventually result in the blocking of the β-catenin destruction complex, thus favoring its nuclear translocation (Figure 2C) [46].

Channel proteins can also contribute to Wnt regulation in a pore-independent manner, such as AQP1, which seems to act as a scaffold for a multiprotein membrane complex directly involved in the regulation of β-catenin. Indeed, recent evidence demonstrated that AQP1 physically interacts with GSK3β, Axin1, and LRP in a multi-complex platform that eventually promotes the destruction of complex activity (Figure 2D). Hence, loss of AQP1 leads to an increase in β-catenin stability, however AQPs seems to act in a context-dependent manner; further details will be debated deep in the following paragraphs; additionally, we remind that Aquaporins (AQPs) are poorly selective channels, gating H_2_O but even small ions [47,48].

## 3. Channels-Wnt-Dependent Signaling Axes in Diseases

Ion channels create and dissipate electrochemical gradients, which are required for cell homeostasis by allowing the passage of water, electrolytes, nutrients, or other substrates through the membranes and transporters. Thus, the involvement of ion channels in pathological conditions is not surprising and has been well described in the literature since the late 1980s’. Then, several pieces of evidence have led the scientific community to use the term “channelopathies” to indicate ion channels dysfunctions associated with pathological conditions [49]. In this context, the purpose of the following sections will be to outline the functional links between ion channels and disease development and progression, specifically analyzing channel-Wnt dependent axes involvement in disorder formation. Particularly, we will focus on the newest findings on ion channels modulation of Wnt signaling, unraveling novel insights in cancer and neurological diseases. The following sections will update the state of the art of the widely reported role of channels in canonical Wnt signaling (see several examples in Table 1, further details can be found in [50]) and will provide a comprehensive and focused point of view on those events.

### 3.1. Channels, Wnt Signaling, and Cancer

Increasing evidence point out the involvement of ion channels belonging to all the five main ion channels family (potassium, chloride, sodium, calcium and non-selective ion channels) in cancer physiopathology [50]. Some of them have been summed up and functionally divided into onco-suppressors and onco-channels in Table 2 and Table 3, specifically focusing on channels involved in regulating Wnt signaling in cancer disease formation and progression.

Moreover, ion channels and Wnt signaling interplay in tumors have been thoroughly and nicely reviewed by Mauss et al. [50].

Thus, the purpose of this section will be to shed light on channels and transporters poorly investigated up to now, especially focusing on AQPs and subcellular channels (i.e., mitochondrial and endoplasmic reticulum ion channels) modulation of Wnt signaling in the context of cancer.

AQPs belong to the major intrinsic protein (MIP) family and consist of ubiquitous tissue and organ-specific channels regulating water flux and small molecules trafficking across cell membranes [48]. Furthermore, several members of the MIP family have been shown to function as ion channels, including AQP0, AQP1, AQP6, while other mammalian AQPs may also serve as ion channels when activated by an appropriate stimulus [63]. Due to the crucial part played by fluid balancing in the regulation of physiological processes, AQPs are reported to be involved in a large variety of cellular mechanisms, including functional changes in cancer cells, mostly linked to stemness maintenance associated with the regulation of canonical Wnt signaling [64]. Although the exact mechanism is still poorly understood, emerging evidence shows that AQPs influence the expression of Wnt downstream signaling molecules, ultimately distinguishing these channels as a possible Wnt-related cancer prognostic marker [64].

Emerging data reveal the existence of an interplay between AQP3 and canonical Wnt signaling in tumors, although contrasting results have been obtained, probably due to tissue-specific expression and function of this channel. Indeed, in 2016, Zhou et al. demonstrated that AQP3 promotes stem-like hallmarks in human gastric cancer cells, enhancing CD44 levels through Wnt/GSK3β/β-catenin signaling pathway. AQP3 overexpression in human gastric cancer cells is associated with an increase in the level of phosphorylated GSK3β inactive form, resulting in a rise of nuclear β-catenin [65]. On the contrary, low levels of AQP3 affect stemness maintenance in human lung cancer stem cells, upregulating the Wnt/GSK3β/β-catenin pathway and Hippo pathway and resulting in altered differentiation and apoptotic processes. Thus, AQP3 influences the expression of canonical Wnt signaling cascade, eventually affecting the proliferation and metastasis formation in lung cancer cells [64].

Furthermore, overexpression of AQP9 is reported to suppress the levels of GSK3β, cyclin D1, and β-catenin in hepatocellular carcinoma cells, enhancing cell apoptosis and inhibiting tumor growth and metastasis formation both in in vitro and in in vivo studies [66], while AQP5 enriches for cancer-driving stem cells in Lgr5+ intestinal cells, the significant sources of cancer following Wnt pathway hyper-activation in mice distal stomach [67].

Concerning intracellular channels, emerging hints suggest that mitochondrial ion channels, which strongly impact mitochondrial function regulation, could be addressed as potential oncological targets against cancer diseases [68,69]. For this reason, research in this field is rapidly expanding, so that the role of some mitochondrial ion channels able to modulate Wnt-signaling in cancerous cells has already been clarified.

Indeed in 2019, Costa et al. demonstrated that treatment with sublethal doses of mitochondrial voltage-gated K^+^ channel Kv1.3 inhibitors (PAPTP and PCARBTP) [69] could downregulate Wnt/β-catenin signaling in vitro in colon cancer cells and in vivo in zebrafish models by reducing mitochondrial ATP production, which, in turn, impairs Ca^2+^ uptake in the ER, thus enhancing ER-stress levels [36].

Along with these findings, *KCNA1*, encoding the voltage-gated K^+^ channel Kv1.1, resulted in being an oncogene strongly expressed in cervical cancer, supporting carcinogenesis by upregulation of Hedgehog, Wnt, and Notch signaling pathways and, eventually, affecting mitochondrial function [70].

Wnt, Hippo, and Notch signaling are all affected by mutations that impair the Sarco-Endoplasmic Reticulum Calcium ATPase (SERCA) pump functional activity in the ER, as demonstrated in the in vivo Drosophila model by Suisse et al. [71]. Concerning Wnt signaling impairment, SERCA activity is crucial for β-catenin/E-cadherin detachment, while Notch and Hippo pathways remain disrupted only at intermediate levels. In this sense, using SERCA inhibitors as cancer therapeutics could be complicated by the differential response of the three oncogenic signaling cascades in cancer disease, suggesting new exciting insights to be taken into account during chemotherapeutic tumor treatment [71].

### 3.2. Ion Channels and Wnt Signaling Regulation in Neurodegeneration and Neurological Diseases

Wnt signaling contribution in the insurgence and the progression of neurodegenerative diseases has only recently been discovered. Because of Wnt fundamental role in orchestrating different neuronal functions in the central nervous system (as, for example, neurogenesis, synapse formation, differentiation, neuroprotection, synaptic plasticity, the integrity of the blood-brain barrier, and biology of microglia), the dysregulation of these pathways has emerged as a crucial player in the pathogenesis of common neurological disorders, such as Parkinson’s disease (PD), Alzheimer’s disease (AD) and other neurodegenerative diseases, like amyotrophic lateral sclerosis (ALS) [72].

In AD, Wnt signaling impairment accelerates the onset of the disease and provokes the development of three AD hallmarks: (i) the production and aggregation of β-amyloid; (ii) tau protein hyper-phosphorylation process that involves GSK3β and Dikkopf 1 (DKK1), and generates neurofibrillary tangles within neurons; (iii) hippocampal-dependent cognitive impairment [73,74,75].

In PD, the evidence of relationships among the early stage of the disease and dysregulated Wnt signaling has been elucidated in the latest years, mainly due to the prominent role of this pathway in the control of midbrain dopaminergic neurons survival and development but also the neuronal rescue and regeneration of PD midbrain [76,77].

The role of Wnt signaling is highly debated in ALS, where the degeneration of motor neurons in the brain cortex could be partly due to the alterations of some Wnt effectors, in particular the dysregulation in β-catenin homeostasis [78,79].

New evidence revealed a direct regulation of the Wnt/β-catenin signaling pathway over channels in neurodegeneration. Specifically, the α7-nicotinic acetylcholine receptor (*CHRNA7*), a Ca^2+^ channel, could represent a potential therapeutic target for neurodegenerative diseases. In AD, its level is drastically reduced in neocortical and hippocampal neurons, together with reducing acetylcholine. It has been demonstrated that activation of canonical Wnt signaling by WNT7A induces *CHRNA7* expression and assembly because its gene promoter contains T-cell factor/lymphoid enhancer factor (TCF/LEF) sites [80,81].

Moreover, in PD, Wnt signaling is a critical effector of *CHRNA7*-induced protection of dopaminergic neurons, suppressing neuroinflammation [82]. McQuate et al., demonstrated a non-canonical Wnt cascade involving WNT5, tyrosine kinase-like orphan receptor 2 (ROR2), G protein and PLC, that decreases K^+^ current and mobilizes intracellular Ca^2+^ from voltage-gated Ca^2+^ channels to upregulate the trafficking of N-methyl-D-aspartate receptors (NMDARs) into synapses, in a SNARE dependent fashion. This mechanism could be of great importance in neuropsychiatric disorders, such as AD, where deregulation of NMDARs occurs [83].

On the other side, only few recent discoveries remark the influence of channels on Wnt signaling. As for cancer and metabolic diseases, even in AD, AQPS has been demonstrated to play an important role in the pathogenesis of the disease. Indeed, the higher expression of AQP1 inhibits Wnt signaling in a mouse AD model, whereas its silencing could activate the pathway, alleviating neuronal apoptosis and improving cognitive function [84].

New insights in the AD non-amyloidogenic pathway that block Aβ production and generate soluble amyloid precursor protein α (APP-α), granting neuroprotection, showed an interplay among NMDARs and Wnt signaling. In particular, NMDAR activation stimulates the secretion of WNT3A, which in turn activates extracellular signal-regulated kinases (ERKs), to elicit A disintegrin and metalloproteinase 10 (ADAM10) —one of the leading α-secretase candidates—responsible for the non-amyloidogenic pathway-expression, likely via β-catenin regulation [75].

### 3.3. Channels, Wnt Signaling in Other Diseases

Several studies show the importance of the interplay between ion channels and Wnt signaling in the insurgence of metabolic disorders. In this respect, Ca^2+^ homeostasis has been demonstrated to be essential for the maintenance of bone marrow mesenchymal stem cells. Considering the Ca^2+^ TRP channel superfamily, it has been elucidated that an impairment in the activity of these channels is related to a decreased Ca^2+^ flux that ultimately downregulates Wnt/β-catenin signaling, leading to the dysregulation of osteogenic differentiation [85]. Another important channel involved in this process is the Ca_V_1.2. Downregulation of Ca_V_1.2 has been found in aging models characterized by an altered osteogenic differentiation that is ultimately caused by the inhibition of the canonical Wnt signaling pathway [86]. Thus, targeting Ca^2+^ channels and homeostasis can be a promising strategy for bone diseases like osteoporosis.

Regarding the role of other cation channels, we can consider polycystins in developing autosomal-dominant polycystic kidney disease (ADPKD) [87]. Polycystins are a class of nonselective cation channels encoded by *PKD1* and *PKD2* genes, involved in the transport of Ca^2+^ inside the cell. Mutations in such genes represent the leading cause of ADPKD development [88]. In particular, Lee et al. suggested the existence of a PKD1/TAZ/Wnt/β-catenin/MYC signaling axis in which is proposed the essential interaction between PKD1 and TAZ. In fact, in the absence of PKD1, TAZ can strongly interact with Axin1, weakening its interaction with β-catenin and inducing the overexpression of c-MYC that lastly leads to the insurgence of renal cystogenesis. Targeting this axis, thus, can be beneficial to decrease ADPKD progression [87].

The importance of AQP1 in ADPKD development has recently been proved, specifically demonstrating that AQP1 deficiency promotes cysts growth through the stimulation of the Wnt signaling cascade [47].

In addition, aquaporins also seem to have an important role in endometriosis. Shu et al. observed that silencing of AQP1, which is upregulated in endometriosis disease, induces the activation of the Wnt signaling pathway that results in a decreased adhesion, invasion, and in the induction of apoptosis of ectopic cells [89].

Finally, regarding anion channels, Lu et al. have demonstrated the importance of the chloride channel ClC-2 in proper blood vessel development in human brain vascular smooth muscle cells (HBVSMCs). The block of this channel activity mediates a decreased Cl^−^ efflux linked to the inhibition of the Wnt/β-catenin pathway. Therefore, this channel can be a promising target for preventing hyperplasia, strokes, and cerebrovascular remodeling [90].

## 4. Conclusions and Future Perspective

This review summarized the major known mechanisms involved in Wnt signaling regulation by channels, ion fluxes, and transporters. Some interesting points emerged from our analysis. Firstly, many pieces of evidence lead to the idea that variation in K^+^ and Ca^2+^ fluxes across cell membrane induced by changes in transmembrane potential can modulate canonical Wnt-signaling [41,51,62]. In addition, this first point is directly linked to our second conclusion: proofs indicate that distinct channels (i.e., Kv 7.1, CFTR, etc.) may have a part in the formation of multiprotein membrane complexes that modulate the correct cytoplasmic-nuclear localization of β-catenin by physical interaction [41,51]. Thus, shedding light on the functional action of membrane polarization and plasma membrane channels upon β-catenin stability would be of great interest for future studies. Furthermore, in the latest years, new evidences on modulation of Wnt signaling by tuning organellar channels are arising, so new hints, as well as new possible modulators, will be proposed in the near future.

The second aim of this review was to elucidate the consequences of the channels-Wnt signaling cascade in different diseases. As clearly reported in the literature, the Wnt/β-catenin pathway is the most associated with channels regulation in cancer disease development and progression [50]. Indeed, the subdivision in onco-channels and onco-suppressors Wnt-regulating proteins certainly corroborates the hypothesis of an ion channels-Wnt signaling axis in cancer. Still, how channels interact with β-catenin or other proteins involved in Wnt cascade sometimes remains elusive and further investigation should be pursued, as for TRPM7, Nav1.5 and CLC-3 onco-channels (in neuroblastoma, oral squamous cell carcinoma and colorectal cancer, respectively) and ClCa1 oncosuppressor (colorectal cancer) [91,92,93,94]. Nevertheless, many data support the idea that inhibition [36,62] or pharmacological activation [41] could be used to properly induce differentiation and tumor aggressiveness arrest via Wnt modulation. Thus, we expect to see an increase in the employ of such treatments in future (pre) clinical studies, possibly using channels levels of expression as prognostic biomarkers due to their contribution to the process of tumorigenesis.

Although the interplay between ion channels and oncogenesis has been unequivocally described, more in-depth investigations about their role in modulating cancerous-unrelated signaling pathways still need to be carried out in other pathologies.

According to the novel findings summarized above, ion channels could be accounted as new Wnt-dependent markers for different pathological conditions, representing invaluable predictive factors for early-onset disease detection and possibly identifying new druggable targets.

## Figures and Tables

**Table 1:** Ion Channels involved in Wnt signaling.

Channels	Ions Gated	Effect	Cell Types and Diseases	Ref.
Kv7.1,	K^+^	Tumor suppression by inactivation of the B catenin in the cytoplasm	Colon and Hepatic carcinoma	[51,52]
Kv11.1	K^+^	Channel activity inactivates β-catenin, preventing nuclear importation	Breast cancer	[41]
CFTR	Cl^-^	Tumor suppressor, CFTR activity contributes to the correct functionality of the complex FZD-LRP-Wnt.	Cystic fibrosis	[53]
TRPV4	Ca^2+^	TRPV4-mediated Ca^2+^ entry from the extracellular space promotes β-catenin activation by AKT modulation	Gastric cancer	[54]
TrpC5	Ca^2+^	TrpC5 overexpression favors the nuclear accumulation of β-catenin by Ca2+	Colorectal cancer	[55]

**Table 2:** Onco-suppressors.

Channels *(ion flux)*	Expression in Cancer	Cancer Type	Effects on Wnt Signaling	Mechanism of Action	Effect on Tumorigenesis	Ref.
**CaCNA2D3** *(Ca^2+^)*	Low	Naso-pharyngeal carcinoma	↓Wnt signaling targets (cyclin D1, c-myc); ↓MMP7 (invasion); ↓ SNAIL (EMT)	↑ intracellular Ca^2+^ flux causes ↑ NLK protein kinase which antagonize canonical Wnt signaling	Overexpression induces mitochondrial-mediated apoptosis and repression of Wnt-dependent invasion, proliferation and EMT.	[56]
**CaCNA2D3** *(Ca^2+^)*	Low	Glioma	↓ Wnt signaling targets (CCND1, c-myc); ↓ MMP7 (invasion); ↓ SNAIL (EMT)	↑ intracellular Ca^2+^ flux causes ↑ NLK protein kinase, which antagonize canonical Wnt signaling	Overexpression induces mitochondrial-mediated apoptosis and repression of Wnt-dependent invasion, proliferation and EMT.	[57]
**CFTR** *(Cl^−^)*	Low	Colorectal cancer; Gastric cancer	↓ Wnt signaling targets	CFTR^−/−^ Apc^min^ mice display ↑Wnt/β-catenin target genes (Ccnd1, CD44, Axin2, Lgr5, Mmp7, Wnt10A and Ptgs2)	CFTR^−/−^ mice developed significantly more tumors in the colon and the entire small intestine and alteration in the intestinal stem cell compartment.	[58]
**Kv7.1** *(K^+^)*	Low	Colorectal cancer	↑β-catenin-E-cadherin interaction	Membrane depolarization prevents cytosolic β-catenin release through β-catenin-E-cadherin complex stabilization	Reduced EMT, cell proliferation, and tumorigenesis	[51]
**Kv11.1** *(K^+^)*	Low	Breast cancer	↑β-catenin-E-cadherin interaction	Membrane depolarization prevents cytosolic β-catenin release through β-catenin-E-cadherin complex stabilization GSK3-β-independent	Represses metastasis formation	[41]

**Table 3:** Onco-channels.

Channels *(ion flux)*	Expression in Cancer	Cancer Type	Effects on Wnt Signaling	Mechanism of Action	Effect on Tumorigenesis	Ref.
**Trp5** *(non-selective Ca^2+^ cation channel)*	High	Colorectal cancer	↑Wnt5a, ↑ Wnt signaling targets (cyclin D1 and c-myc)	↑ Trp5 increase Ca^2+^ influx in the cell, which induces ↑Wnt5a and ↑ nuclear β-catenin levels	Channel upregulation reduces colorectal cancer differentiation and stemness through Ca2^+^-Wnt5a	[59]
**Trp5** *(non-selective Ca^2+^ cation channel)*	High	Colorectal cancer	β-catenin stabilization	Reduction in levels of the channels reduces Ca^2+^ influx lowering β-catenin stability	Silencing of the channel reduces colorectal cancer tumorigenesis and ameliorates 5-Fluorouracil chemoresistance	[55]
**TRPM4** *(non-selective Ca^2+^ cation channel)*	High	Prostate cancer	↑β-catenin stabilization	↑ GSK3β and AKT1 phospho-inactive form through Ca^2+^ flux	Tumor proliferation and progression	[60]
**TRPV2** *(non-selective Ca^2+^ cation channel)*	High	Esophageal squamous cell carcinoma	↑ Wnt signaling	Increased Ca^2+^ flux results in ↑ Wnt signaling. The precise mechanism of action still to be determined.	Promotes proliferation, invasion, and angiogenesis	[61]
**BK_Ca_** *(K^+^)*	High	Breast cancer	↑β-catenin stabilization	Modulation of transmembrane depolarization regulates phospho-AKT and, in turn, β-catenin stability	Pharmacological inhibition of the channel (*Ibtx*) reduces anchorage-dependent growth and tumorigenesis	[62]
